# mTOR inhibition affects Yap1-β-catenin-induced hepatoblastoma growth and development

**DOI:** 10.18632/oncotarget.26668

**Published:** 2019-02-19

**Authors:** Laura Molina, Hong Yang, Adeola O. Adebayo Michael, Michael Oertel, Aaron Bell, Sucha Singh, Xin Chen, Junyan Tao, Satdarshan P.S. Monga

**Affiliations:** ^1^ Division of Experimental Pathology, Department of Pathology, University of Pittsburgh School of Medicine, Pittsburgh, PA, USA; ^2^ Medical Scientist Training Program, University of Pittsburgh School of Medicine, Pittsburgh, PA, USA; ^3^ Department of Medical Ultrasonics, The First Affiliated Hospital of Guangxi Medical University, Guangxi, China; ^4^ Department of Biomedical Engineering, Georgia Institute of Technology, Atlanta, GA, USA; ^5^ Pittsburgh Liver Research Center, University of Pittsburgh Medical Center and University of Pittsburgh School of Medicine, Pittsburgh PA, USA; ^6^ Department of Bioengineering and Therapeutic Sciences and Liver Center, University of California, San Francisco, CA, USA; ^7^ Division of Gastroenterology, Hepatology and Nutrition, Department of Medicine, University of Pittsburgh School of Medicine, Pittsburgh, PA, USA

**Keywords:** pediatric liver tumor, Wnt signaling, mTOR pathway, beta-catenin, YAP1

## Abstract

Hepatoblastoma (HB) is the most common pediatric liver malignancy. Around 80% of HB demonstrate simultaneous activation of β-catenin and Yes-associated protein 1 (Yap1). The mechanism by which these signaling pathways contribute to HB pathogenesis remain obscure. Recently, mTORC1 activation was reported in human HB cells and in a murine HB model driven by β-catenin and Yap1. Here, we directly investigate the therapeutic impact of mTOR inhibition following HB development in the Yap1-β-catenin model. HB were established by hydrodynamic tail vein injection of Sleeping Beauty transposase and plasmids coding for ΔN90-β-catenin and S127A-Yap1. Five weeks after injection, when HB were evident, mice were randomized into Rapamycin diet-fed or basal-diet-fed groups for 5-weeks. Tumor growth was monitored via ultrasound imaging and mice in both groups were euthanized after 5-weeks for molecular analysis. Transcriptomic analysis showed a strong correlation in gene expression between HB in the Yap1-β-catenin model and HB patient cohorts. Rapamycin treatment decreased HB burden, almost normalizing liver weight to body weight ratio. Ultrasound imaging showed reduction in tumor growth over the duration of Rapamycin treatment as compared to controls. Majority of HB in the controls exhibited crowded fetal or embryonal histology, while remnant tumors in the experimental group showed well-differentiated fetal morphology. Immunohistochemistry confirmed inhibition of mTORC1 in the Rapamycin group. Thus, Rapamycin reduces HB in a clinically relevant model driven by β-catenin and Yap1, supporting use of mTORC1 inhibitors in their therapy. We also show the utility of standard and 3D ultrasound imaging for monitoring liver tumors in mice.

## INTRODUCTION

Hepatoblastoma (HB) is the most common pediatric liver cancer and is commonly diagnosed in the first few years of life [1]. Despite being a rare cancer, the annual incidence of HB has gradually increased over the past three decades [2]. Most cases of HB appear to be sporadic, but some are associated with genetic abnormalities and malformations, such as in cases with Beckwith-Wiedemann syndrome and familial adenomatous polyposis [3, 4]. Premature babies with low birth weight are also at a greater risk of developing HB [1, 5]. At present, surgical resection along with chemotherapy remains the curative strategy for HB and offers the only realistic chance of long-term disease-free survival [6]. Investigating the genetic and molecular origins of HB will provide better understanding of the disease and identify novel therapeutic approaches.

HBs arise from hepatoblasts, fetal progenitor cells of the liver, and are categorized by histological subtyping based on the level of cell differentiation [7]. Despite the rarity of these tumors, several studies have used small patient cohorts to characterize genomic and transcriptomic alterations to identify tumor drivers, of which the most common is β-catenin. β-catenin is a downstream effector of the Wnt pathway and plays a critical role in hepatoblast proliferation and hepatocyte differentiation in normal hepatic development [8]. In about 60-70% cases of HB, deletion or missense mutations have been identified in the CTNNB1 gene encoding for β-catenin [9, 10]. These mutations impair the phosphorylation and degradation of β-catenin, leading to constitutively active β-catenin [11].

We recently showed many cases of sporadic HBs to exhibit nuclear localization of β-catenin and Yes-associated protein 1 (Yap1), which is a major effector of the Hippo signaling pathway playing a key role in regulating liver size and liver cell differentiation [12, 13]. Based on this evidence, we developed a unique mouse model of HB driven by co-activation of Yap1 and β-catenin [12]. Upon co-delivery of Sleeping-Beauty (SB) transposase and plasmids containing mutant DN90-β-catenin and S127A-Yap1 to the liver by hydrodynamic tail vein injection (HTVI), a small fraction of hepatocytes stably co-express the two oncogenes, which resulted in permanent transformation. This Yap1-β-catenin model results in rapid development of HB in mice allowing investigation of biology, mechanisms and therapies.

Several studies have also shown the importance of mammalian target of Rapamycin (mTOR) activation in HB tumor growth [14–16]. Based on this evidence, we hypothesized that pharmacologic mTORC1 inhibition using Rapamycin (Sirolimus), an FDA-approved agent indicated for prevention of transplant rejection, oncology and orphan conditions like lymphangioleiomyomatosis, would significantly impair HB tumor growth *in vivo* [17, 18]. Five weeks after establishing Yap1-β-catenin driven HB using SB-HTVI, we monitored tumor growth and development using non-invasive 2D and 3D ultrasound (US) imaging to evaluate changes in tumor burden in the same mice over time, producing a more accurate representation of the effects of Rapamycin while reducing the number of animals used for the study. Additional analysis and validation of US imaging was done after 5-week treatment with Rapamycin. Our results show that Rapamycin significantly reduces HB burden *in vivo*, by affecting diminishing mTORC1 activation, proliferation and altering histology of HB from an embryonal to a well-differentiated fetal subtype. This study supports the clinical use of Rapamycin for a subset of HB driven by Yap1-β-catenin co-activation.

## RESULTS

### S127A-Yap1-ΔN90-β-catenin driven murine hepatoblastoma tumors show significant correlation of gene expression to independent cohorts of patient hepatoblastoma tumors and are associated with more proliferative HB subtypes

**Figure 1:**
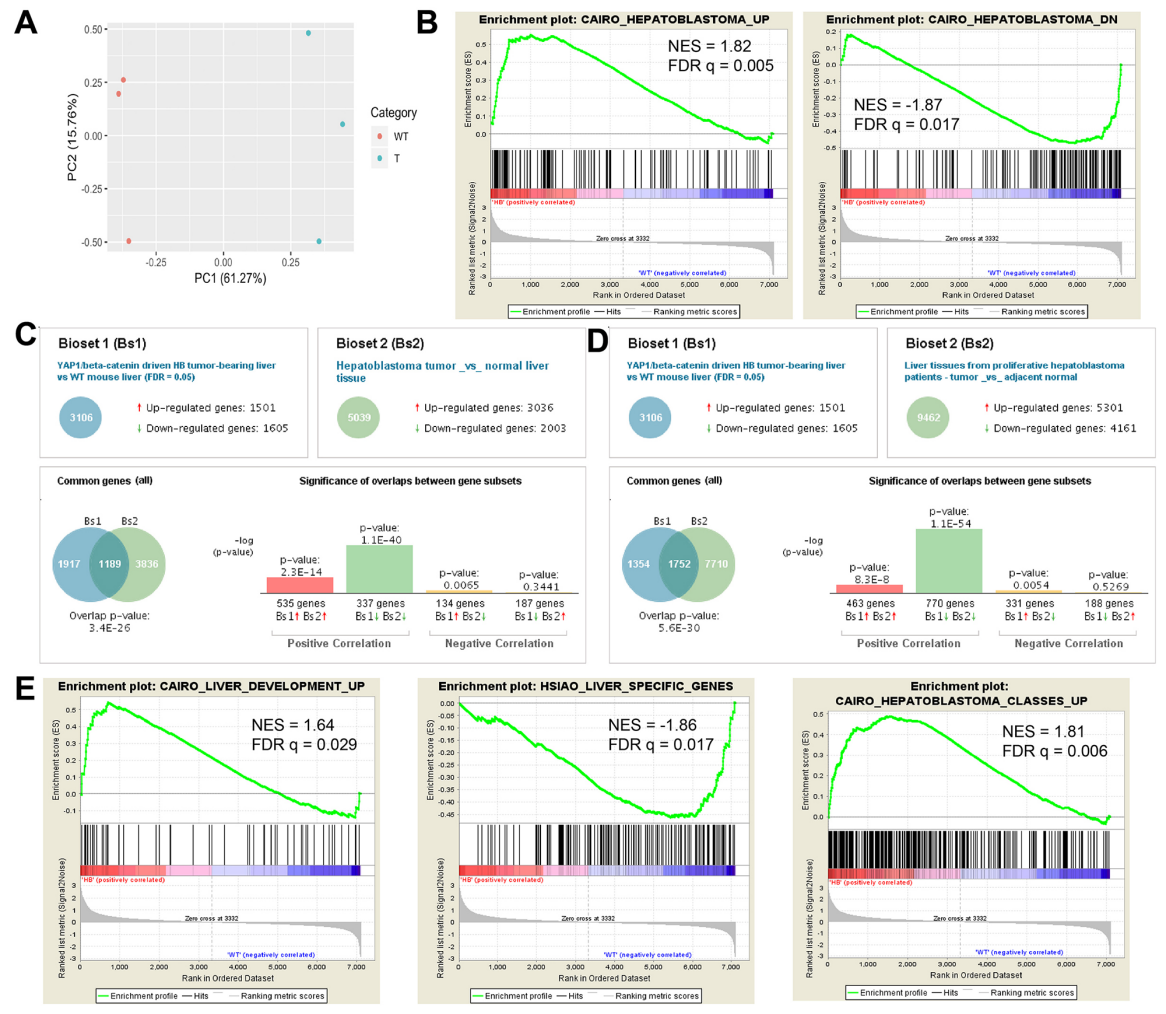
HB occurring in the Yap1-β-catenin model show similarity to HB in patients by transcriptomic analysis **(A)** Principal component analysis (PCA) plot derived from Affymetrix microarray gene expression analysis shows that wildtype (WT) and HB tumor-laden (T) liver samples cluster separately along the PC1 axis, with PC1 explaining 61.27% of the variance in the data. **(B)** Gene Set Enrichment Analysis for gene sets upregulated (Cairo_Hepatoblastoma_Up) or downregulated (Cairo_Hepatoblastoma_Down) in patient hepatoblastoma tumors shows significant enrichment of HB genes in our mouse model [31]. **(C-D)** BaseSpace Correlation Engine software was used to determine the overlap in the set of differentially expressed genes in our HB tumors relative to WT liver (Bioset 1) with gene expression data sets enriched in HB tumors from independent patient cohorts published by Cairo *et al* (C, Bioset 2) and Hooks *et al* (D, Bioset 2) [31, 32]. **(E)** GSEA analysis shows significant enrichment in murine HB tumors for genes expressed in early liver development (Cairo_Liver_Development_Up) and for genes expressed in a proliferative subclass of HB patient tumors (Cairo_Hepatoblastoma_Classes_Up), while genes enriched in mature adult liver tissue are significantly enriched in WT over HB samples (Hsiao_Liver_Specific_Genes). NES, normalized enrichment score. FDR, false discovery rate.

We first aimed to determine the clinical relevance of our mouse model of HB driven by Yap1 and β-catenin activation by comparing gene expression patterns in murine HB to available gene expression data sets derived from human HB tumors. To do this, we used Affymetrix microarray to compare gene expression of livers with late-stage HB tumors (n = 3) induced by Yap1-β-catenin SB-HTVI to WT murine liver (n = 3). Principal component analysis shows that the HB tumors mostly cluster together and distantly from the WT liver tissue, as expected (Figure 1A). We then compared our data set with previously published gene expression data of human HB patient cohorts using two different methods. Using Gene Set Enrichment Analysis (GSEA) software, we determined that mouse HB tumors showed a significant enrichment of genes upregulated in a cohort of 25 human HB tumors studied by Cairo *et al*, as well as a significant negative correlation with genes downregulated in human HB tumors (Figure 1B) [19, 20]. We next identified differentially expressed genes using a false discovery rate of 0.05, and uploaded our gene set into BaseSpace Correlation Engine, which compares the dataset with a large library of curated gene sets from the literature [21]. Notably, we identified highly significant overlap between our HB data set and human HB tumor data sets from both the Cairo *et al* patient cohort (Figure 1C) as well as an independent HB patient cohort profiled by Hooks *et al* (Figure 1D) [19, 22]. The results show a strong positive correlation among upregulated and downregulated genes in all three data sets. This data further strengthens the correlation in gene expression patterns between our HB mouse model and patient HB tumors, supporting our use of this model for further preclinical investigation.

Through GSEA analysis, we also identified a significant enrichment of genes expressed in early liver development (embryonic days 11.5-12.5) as compared to later developmental stages, while genes characteristically expressed in mature adult hepatocytes were significantly enriched in WT samples as opposed to HB tumors (Figure 1E) [19, 23]. Previously, Cairo *et al* had distinguished two classes of HB tumors based on a 16 gene signature correlated with tumor differentiation state and patient prognosis, and identified a subclass of more highly proliferative tumors associated with less well-differentiated tumor types and overall decreased survival [19]. Notably, we identified that genes significantly upregulated in this subclass of proliferative patient HB tumors relative to more well-differentiated HB tumors were also significantly enriched in our mouse model of HB (Figure 1E). This data is consistent with the enrichment of poorly differentiated hepatoblast-like tumor cells in the mouse HB liver samples and suggests that our tumor model exhibits features of more aggressive HB tumors.

### Mice treated with Rapamycin show significantly decreased hepatoblastoma tumor burden

**Figure 2:**
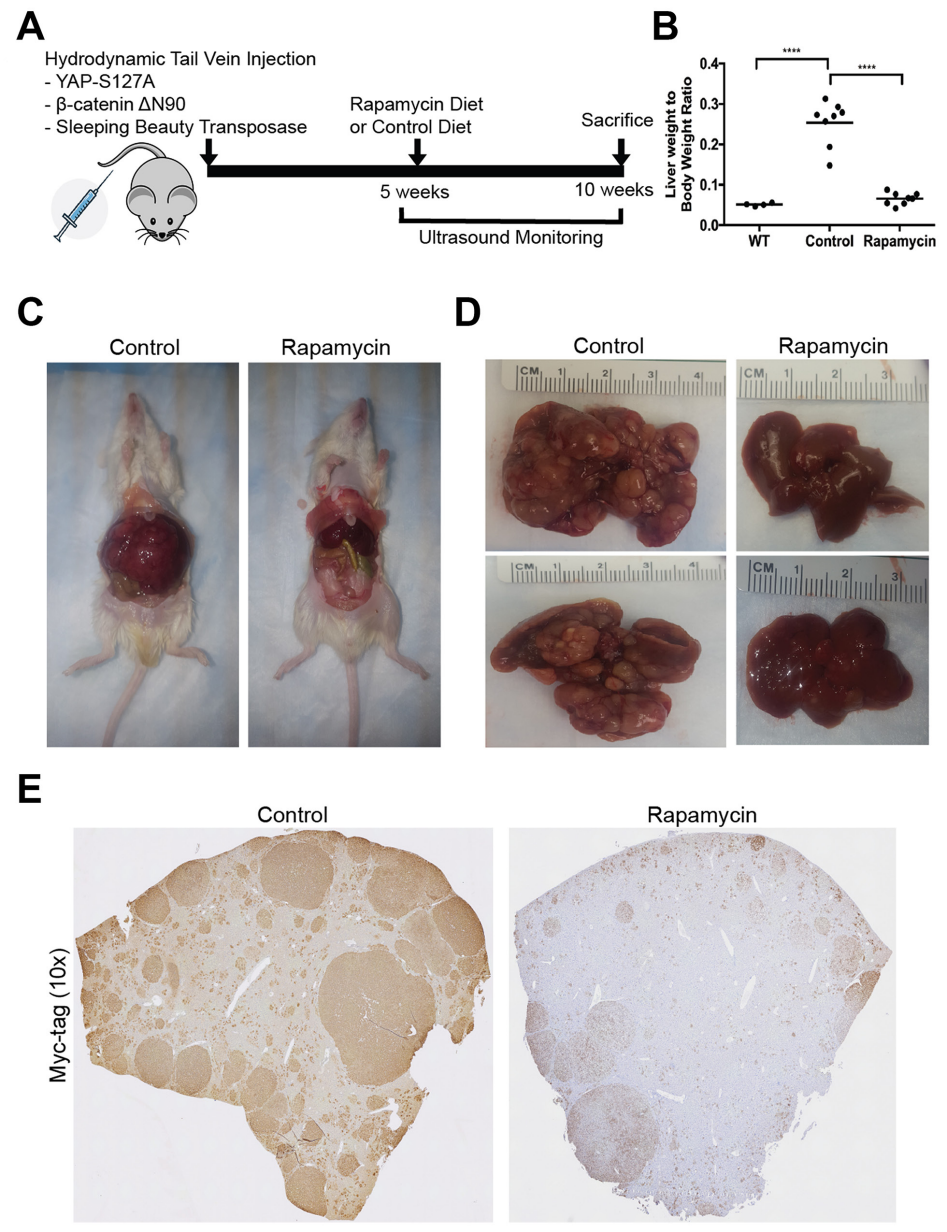
A notable decrease in tumor burden in the Yap1-β-catenin mouse model following Rapamycin treatment **(A)** Schematic detailing the generation of Yap1-β-catenin HB mouse model and randomization to Rapamycin or control diet. **(B)** Liver weight to body weight ratio of wild type (WT) mice which are historical controls, mice 10 weeks post-HTVI on control diet (Control), and mice 10 weeks post-HTVI treated with 5 weeks of Rapamycin diet (Rapamycin). **(C)** Gross images showing excessive liver growth due to tumor formation in control mice post-HTVI, which does not occur in Rapamycin treated mice. **(D)** Gross pathology images show that control mice have significant tumor burden, while Rapamycin-treated mice show few, small tumors and appear mostly normal. **(E)** Representative tiled images of one liver lobe comparing tumor burden in control (160 tiles) and Rapamycin treated (110 tiles) mice using immunohistochemistry to target Myc-tag (representing exogenous, mutant β-catenin).

We next used our clinically relevant HB model to address the potential therapeutic efficacy of mTORC1 inhibition to decrease HB tumor growth. We used the SB-HTVI system to induce hepatoblastoma tumor formation driven by mutant Yap1-S127A and β-catenin-ΔN90 in 5-week old FVB mice. As reported previously as well, at 5 weeks, small tumors are already present [12]. At this stage, we began treating half of the mice with Rapamycin through diet as described in the Methods, and used ultrasound (US) imaging to monitor tumor growth in control and treatment groups (Figure 2A). By 10 weeks post-HTVI, control mice exhibited severe abdominal distension reflecting extensive tumor burden, requiring euthanasia. At this time-point, liver weight to body weight ratio (LW/BW) showed a dramatic increase in control mice (around 25%) versus around 5% in normal wild-type (WT) mice reflecting a profound tumor burden (Figure 2B). In contrast, mice treated with 5 weeks of Rapamycin diet showed a significant decrease LW/BW (around 5.5%) compared to the control group, and was insignificantly different from WT mice, thus displaying a dramatically lower tumor burden compared to the control mice (Figure 2B). Indeed tumor-laden livers were clearly visible in the abdomens of controls but not in Rapamycin-treated group (Figure 2C). Representative gross pathology images showed an abundance of large and small tumors in the control group, while Rapamycin treated livers showed very few and notably smaller nodules, and largely normal appearance (Figure 2D). Immunohistochemistry against Myc-tag, which labels exogenous ΔN90-β-catenin, to compare the distribution of transformed cells in control vs Rapamycin treated groups, was performed next. Figure 2E shows representative tiled images of control and Rapamycin treated mice. Control mice 10 weeks post-HTVI showed abundant large and small Myc-tag positive HB tumor nodules occupying the majority of the liver lobe, while Rapamycin treated group showed predominantly normal liver parenchyma with occasional small tumor nodules and small clusters of transformed cells. In addition, nuclear Yap1 was evident in all HB in the control group by immunohistochemistry while Rapamycin treatment led to notably smaller HB which still showed nuclear Yap1 (Supplementary Figure 1A). Altogether, this data showed Rapamycin treatment led to a dramatic decrease on growth and development of HB in the Yap1-β-catenin mice.

### 3D ultrasound verifies a reduction in tumor burden by Rapamycin in the Yap1-β-catenin model

**Figure 3:**
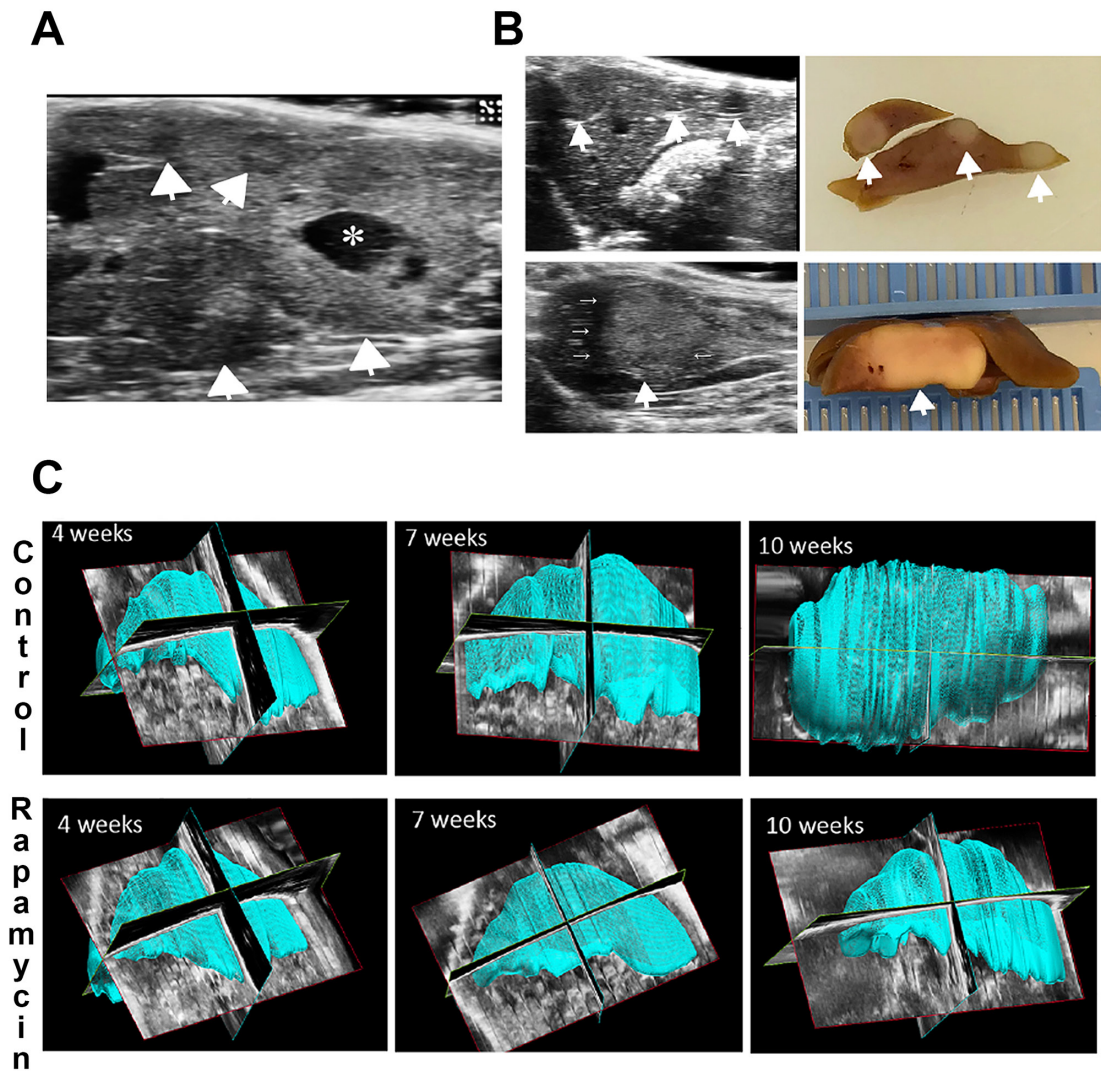
Ultrasound and ultrasound-based 3D to detect HB and liver volume in the Yap1-β-catenin model **(A)** Representative ultrasound image of a control mouse showing multiple large, focal, well-circumscribed HB tumors (arrowheads) as well as a large area of necrosis (asterisk). **(B)** Representative ultrasound images are shown alongside the gross pathology images of the same liver. Both pairs of images represent control mice 10 weeks post-HTVI showing multiple HB tumors (arrowheads). Small arrows highlight an acoustic halo around the largest tumor. **(C)** Representative reconstructed 3D liver images used to calculate total liver volume for control and Rapamycin-treated mice at multiple time points including 4, 7, and 10 weeks post-HTVI.

We also used standard and 3D ultrasound monitoring to quantify tumor growth over time in both groups, as described in the Methods, and address effect of Rapamycin on tumor growth. The goal was to determine whether US monitoring could effectively quantify liver tumor burden in mice over time and hence limit the number of mice needed for studies. A notable tumor burden in the form of multiple hypo- or hyperechoic, round, well-circumscribed, focal lesions, was clearly evident in a representative US image of a control mouse liver, 10 weeks post SB-HTVI, with the largest lesions showing areas of necrosis (Figure 3A). As a proof of concept, the US image could be matched with gross pathology showing pale, round lesions distinct from the surrounding liver as shown in examples from two control mice 10 weeks post-HTVI (Figure 3B). Additionally, the largest tumors also showed a non-echoic acoustic halo on US (Figure 3B, lower panels).

While standard US imaging can detect the presence of tumors with diameter of at least 0.5 mm, it lacks a cohesive view of the whole liver as opposed to CT or MRI scans to directly compare tumor burden from mouse to mouse. To address this limitation, we used 3D-US imaging to measure quantitative volumetric parameters for each mouse weekly, starting at 4 weeks post-HTVI for both the control and Rapamycin treated group. With a 20MHz probe stabilized on a mechanical arm, successive transverse images of the whole liver were obtained and compiled to create a 3D-scan of the liver. Prior to Rapamycin treatment, at 4 weeks after HTVI of plasmids, livers appeared comparable in both groups, as the tumors present at this time point were small and dispersed throughout the liver (Figure 3C). At this time, most tumors were smaller than the limit of detection of the standard US imaging. At 7 weeks, control livers began to appear larger than the Rapamycin treatment group, and by 10 weeks, control livers showed a dramatic loss of the normal liver shape due to the irregular expansion of the tumors also visible as an irregular surface contour (Figure 3C). The livers in the mice treated with Rapamycin showed normal liver size and shape with only a few surface irregularities, if at all (Figure 3C).

**Figure 4:**
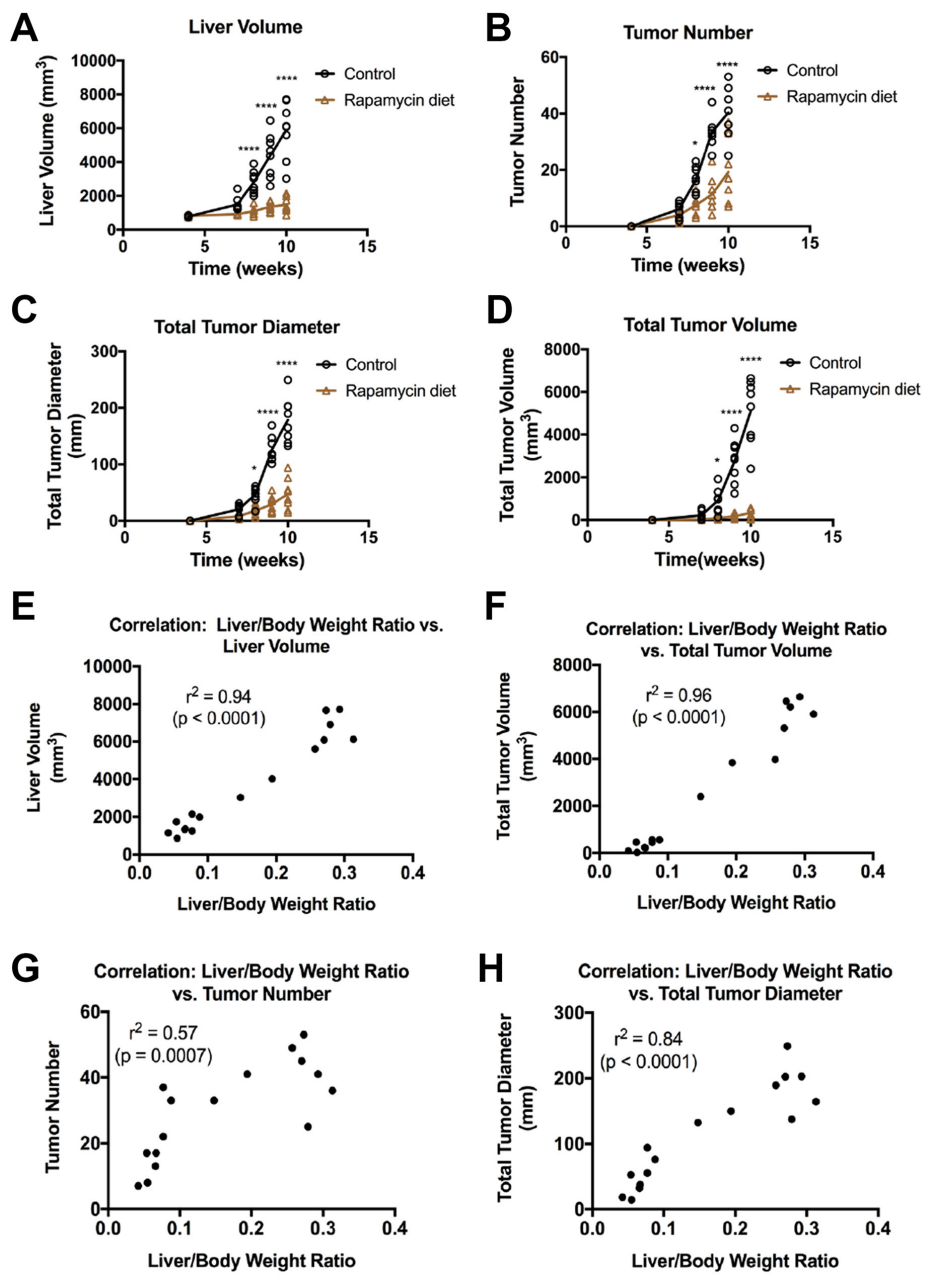
Effect of Rapamycin on liver volume and tumor number, volume and diameter in the Yap1-β-catenin HB mouse model **(A-D)** 3D-US imaging was used to approximate the total liver volume (A), number of tumors in each liver (B), total tumor diameter (C), and total tumor volume (D) of each mouse from 4 to 10 weeks post-HTVI, showing a dramatic increase in tumor burden in control mice, which is significantly stunted in mice fed Rapamycin from weeks 5-10 post-HTVI (^*^, p < 0.05; ^****^, p < 0.0001). **(E-H)** At 10 weeks post-HTVI, mice were sacrificed, and for each mouse the liver weight to body weight ratio (LW/BW) was compared with the 3D-US parameters from the same time point including liver volume (E), total tumor volume (F), tumor number (G), and total tumor diameter (H). The correlation between LW/BW and each parameter was determined; Pearson’s r^2^ correlation coefficient are shown on each graph.

Next, a radiologist (H.Y.) evaluated each scan frame-by-frame to quantify tumor number, tumor diameter, total tumor volume, and total liver volume for each mouse to compare the time course of tumor growth in both controls and Rapamycin-treated groups. Some representative frames used to assess such parameters are included (Supplementary Figure 1B). A detailed recording of these parameters is included in Table 1. Control mice showed a steady increase in liver volume starting at 7 weeks post-HTVI (Figure 4A), and the combined increase in the tumor numbers (Figure 4B) and tumor diameter (Figure 4C) over time resulted in an exponential increase in total tumor volume (Figure 4D). In contrast, mice treated with Rapamycin showed only a small increase in total liver volume (Figure 4A) and these parameters diverge significantly from control mice after just 3 weeks of Rapamycin treatment (Figure 4A). Significant decreases in tumor number and tumor diameter were also visible in Rapamycin-treated versus control mice at all tested times (Figure 4B, 4C). Thus, the final tumor volume in Rapamycin treated mice was extremely small and significantly lower than controls showing its efficacy in reducing overall tumor burden in the Yap1-β-catenin model of HB (Figure 4D). Intriguingly, the 3D-US data showed an upward trend in tumor number and size over time in the Rapamycin treated group, showing that Rapamycin delayed but did not completely abolish HB tumor growth in this model.

**Table 1:** Liver volume, tumor number, total tumor diameter, and total tumor volume in control and treated groups at 4,7,8,9,10 weeks after injection

	Liver volume (mm^3^)		Tumor number		Total tumor diameter (mm)		Total tumor volume (mm^3^)	
	control	treated	control	treated	control	treated	control	treated
**4w**	799.8±51.3	829.3±51.6	0	0	0	0	0	0
**7w**	1492.9±416.5	930.9±74.1	6±3	4±2	20.3±9.2	7.9±4.6	218.7±185.7	22.1±23.2
**8w**	2816±664.1	1104.4±236.3	16±5	8±4	56.5±17.5	21.3±16.4	901.9±477.9	79.2±58.7
**9w**	4386.1±1305.3	1371.2±289.1	33±5	11±6	125.7±23.0	29.2±14.9	2759.0±1006.6	178.7±107.3
**10w**	5893.2±1661.6	1475.9±441.3	40±9	19±11	178.5±39.8	47.6±27.7	5093.8±1525.1	330.4±208.3

To evaluate the sensitivity and accuracy of 3D-US scans, we next determined the correlation between the four volumetric US parameters measured at 10 weeks post-HTVI and the liver weight to body weight ratio of each mouse, which is the standard and surrogate for representing the liver tumor burden in a mouse model. Indeed, total liver volume and total tumor volume determined by 3D-US, showed a very strong correlation with liver weight to body weight ratio across a broad dynamic range and hence could have an application as a surrogate measure to compare tumor burden in mice prior to sacrifice (Figure 4E, 4F). Total tumor diameter showed a strong correlation but with increased variability, while total tumor number showed only a moderate correlation, most likely due to the wide range of tumor sizes in both the control and Rapamycin-treated groups (Figure 4G, 4H). This data shows that 3D-US parameters can be used to measure tumor burden and can reduce the number of mice used for experiments while providing important temporal information about tumor growth *in vivo*.

### Rapamycin treatment profoundly affects HB development

**Figure 5:**
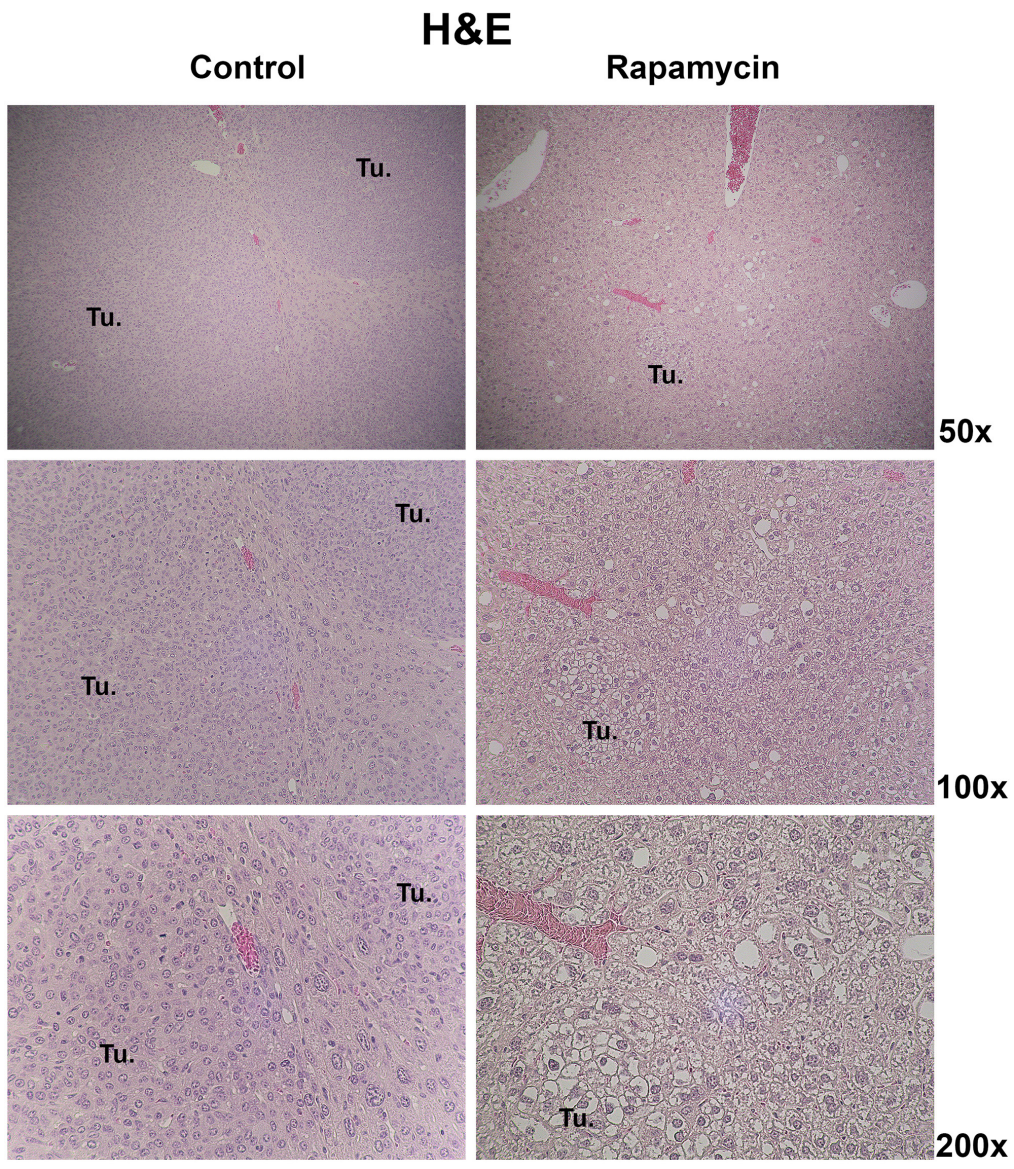
Histology of HB in the Yap1-β-catenin with and without Rapamycin treatment Representative images of H&E staining showing the predominant histological features of HB tumors in control mice and Rapamycin-treated mice 10 weeks post-HTVI. (Tu. – Tumor foci).

While the overall tumor burden was notably decreased in the Rapamycin-treated group, we next wanted to address if Rapamycin treatment also led to any differences in tumor histology. Hematoxylin and Eosin (H&E) staining was performed on liver samples from both groups (Figure 5). At 10 weeks after HTVI, the histology of the tumors occurring in the control group was that of crowded fetal or embryonal HB (Figure 5). In the Rapamycin treated group, the overall tumor burden was dramatically lower but none of the remnant tumors showed any embryonal or crowded fetal histology. Instead, the small tumors evident in this group were composed of well-differentiated cells and histology was reminiscent of well-differentiated fetal HB (Figure 5). The tumor cells were composed of hepatocytes with clear cytoplasm.

**Figure 6:**
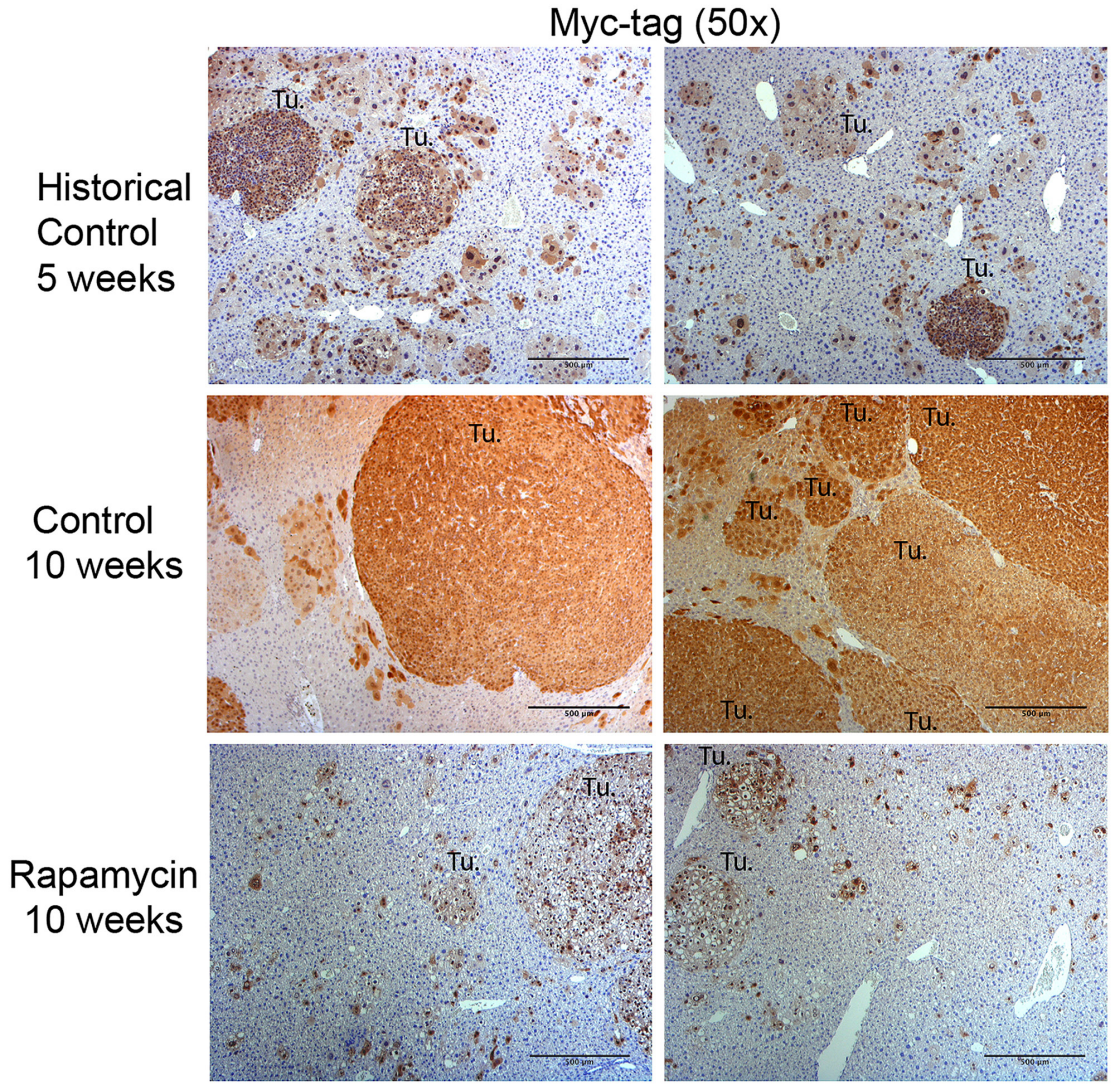
Immunohistochemistry for Myc-tag in the Yap1-β-catenin HB mouse model with and without Rapamycin treatment Immunohistochemistry for Myc-tag (representing exogenous, mutant β-catenin) was used to distinguish tumor cells in mice 5 weeks post-HTVI (historical controls), 10 weeks post-HTVI (control), and 10 weeks post-HTVI treated with 5 weeks of Rapamycin diet (Rapamycin). Representative images from control mice show abundant HB tumors at 10 weeks, most of which demonstrated embryonal histology. While Rapamycin treated mice show a tumor burden similar to that observed in historical control mice at 5 weeks after HTVI, the histology of Rapamycin-treated tumors is notably altered and represents mostly well-differentiated fetal HB. (Tu.- Tumor foci; Scale bar: 500 μm).

We next performed immunohistochemistry for Myc-tag that represents the exogenous β-catenin since Myc-tag is linked to the ΔN90-β-catenin plasmid. Using the historical control mice, we show that at 5 weeks post-HTVI mice develop small tumor nodules with diameters of around 0.5 mm containing clusters of transformed cells (Figure 6). The predominant histology of cells clustering as microscopic tumor foci was reminiscent of HB, as published previously [12]. At 10 weeks post-HTVI, the livers in the control group are packed with abundant HB exhibiting embryonal or crowded fetal histology (Figure 6). Rapamycin-treated mice showed an overall dramatic reduction in numbers and size of tumors, with tumor size and distribution almost similar to that observed in control mice at 5 weeks post-HTVI, suggesting that a notable cytostatic role of Rapamycin (Figure 6). Importantly, the remnant tumors in the Rapamycin-treated group were still composed of Myc-tag-positive cells but the histology was indicative more of a well-differentiated HB which were smaller and composed of clear but small hepatocytes (Figure 6). Likewise, as shown previously, nuclear Yap1 was evident in all HB in the control group by immunohistochemistry while Rapamycin treatment led to notably smaller HB which still showed nuclear Yap1 (Supplementary Figure 1A). Thus, Rapamycin treatment differentially diminished the growth and survival of the HB with predominant crowded fetal or embryonal-like histology, while the smaller foci composed of more differentiated HB resembling fetal form persisted [12].

**Figure 7:**
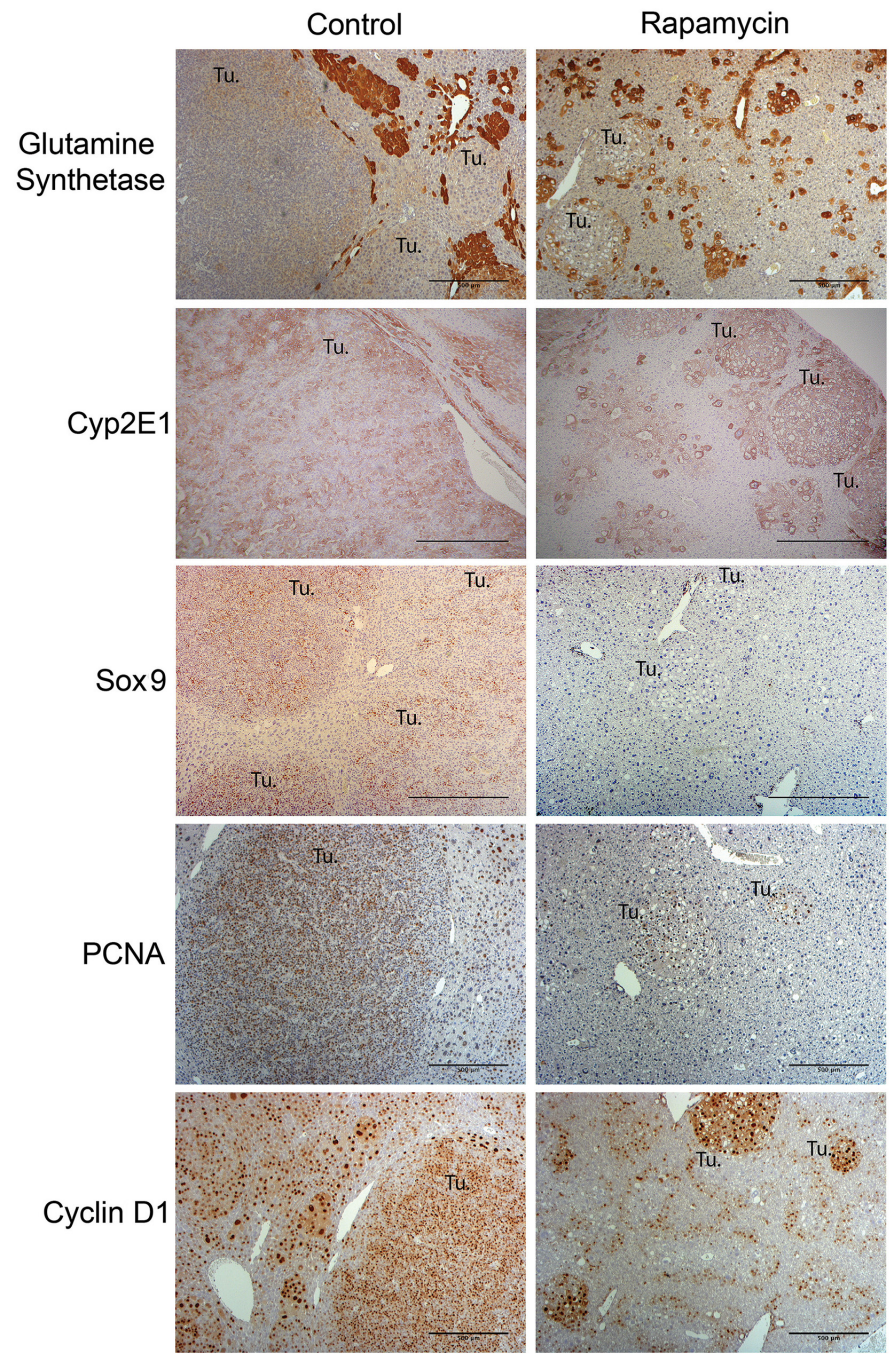
Molecular characterization of HB in the Yap1-β-catenin HB mice treated with Rapamycin as compared to the untreated group Representative immunohistochemistry images (50x) assessing differentiation and proliferation of HB with and without Rapamycin treatment. Representative immunostaining for Glutamine Synthetase (GS) shows that HB tumors in both the control and Rapamycin treatment group are negative for this marker (50x). Immunostaining for Cyp2E1 (50x) shows mixed expression levels in control HB tumors but high levels of expression in Rapamycin-treated tumors. Immunostaining for Sox9 (50x) shows that control HB tumors are strongly positive while Rapamycin treated tumors are mostly negative. Representative immunohistochemistry images (50x) for cyclin-D1 and PCNA staining compare control versus Rapamycin treatment group showing comparable intratumoral proliferation despite a dramatic difference in tumor sizes between the two groups. (Tu.- Tumor foci; Scale bar: 500 μm).

Glutamine synthetase (GS) has been used to distinguish a more differentiated fetal from an undifferentiated embryonal HB [9]. We have also reported previously that HB that occur in Yap1-β-catenin model are only transiently GS-positive at the earliest stages of tumorigenesis [12]. Indeed, HB in the control group after 10 weeks of HTVI were GS-negative (Figure 7). Most of the tumors in the Rapamycin-treated group also continued to show GS-negative nodules despite the resemblance to a fetal HB like morphology (Figure 7). However, while HB tumors in the control group showed a mixed expression pattern of Cyp2E1 (a pericentrally zonated gene associated with well-differentiated tumors), Rapamycin-treated tumors were strongly positive for Cyp2E1 (Figure 7) [19]. We also evaluated the expression of biliary marker Sox9; expression of biliary markers has been associated with less differentiated tumor types [9, 19]. Consistent with histological observations, the crowded fetal and embryonal tumors in the control group were strongly positive for Sox9, but Rapamycin-treated tumors showed significantly decreased expression of Sox9 and several remnant HB were often Sox9 negative (Figure 7). Altogether, these observations suggest that alterations in cell morphology following Rapamycin treatment are also associated with increases in some molecular hallmarks of tumor cell differentiation.

### Rapamycin treatment decreased tumor cell proliferation

To directly address the biological effect of mTORC1 activation and its inhibition by Rapamycin, we next assessed tumor proliferation in both control and experimental group. Cyclin-D1, an important regulator of G1 to S phase cell cycle transition, was tested by immunohistochemistry. In both the control and Rapamycin treated group, the tumor cells continued to be positive for cyclin-D1 irrespective of the size of the tumor foci (Figure 7). Proliferation was next assessed by PCNA immunohistochemistry. Like Cyclin-D1, tumor cells in both the control as well as the Rapamycin-treated groups were positive for PCNA (Figure 7). Thus, while a clear decrease in tumor numbers, size and histology was apparent after 5 weeks of Rapamycin treatment, the remnant tumor foci were PCNA staining even in the Rapamycin-treated group.

### Rapamycin treatment affects mTORC1 signaling in the Yap1-β-catenin model of HB development

**Figure 8:**
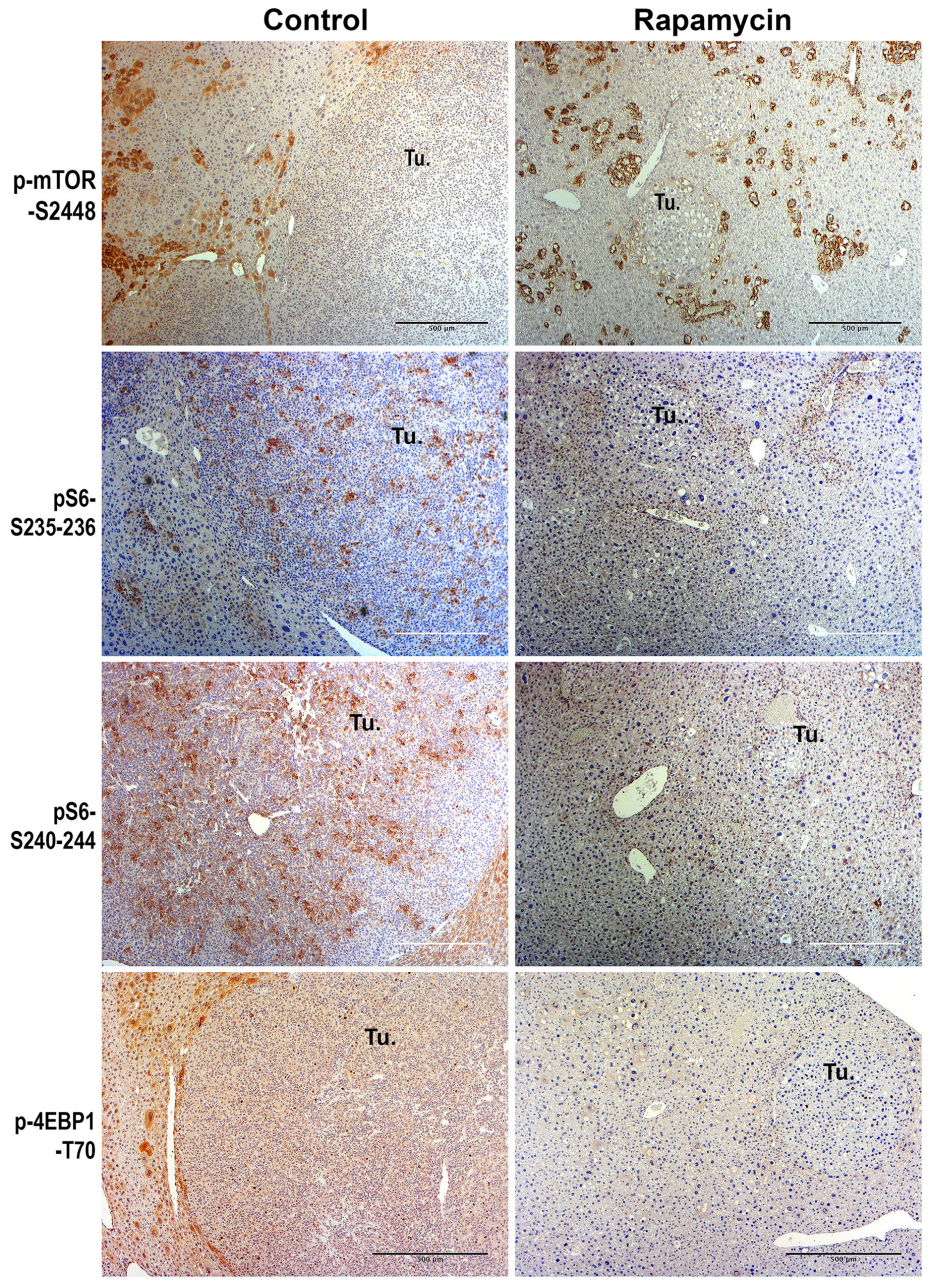
Immunohistochemical analysis of mTORC1 signaling in the HB in the Yap1-β-catenin mice treated with Rapamycin versus no treatment Representative immunohistochemistry images (50x) assessing mTORC1 signaling in HB in the control versus Rapamycin treatment group. HB were negative for p-mTOR-S2448 in both groups. HB were positive for p-S6 ribosomal protein (pS6)-S235-236 and pS6-S240-244, and p-4EBP1-T70 in the control group but remnant tumors were completely negative for all these markers in the Rapamycin-treated group. (Tu.- Tumor foci; Scale bar: 500 μm).

To address if Rapamycin effectively reduced signaling in HB, we next performed immunohistochemistry on the livers from the control and treatment group for downstream effectors of mTORC1. Intriguingly the HBs observed in the control mice at 10 weeks did not show any notable staining for phospho-mTOR-S2448, an indicator of active-mTORC1, by immunohistochemistry (Figure 8). Remnant tumors in the Rapamycin-treated group also stained negative for p-mTOR-S2448 (Figure 7). Only occasional small foci composed of a cluster of a few cells were p-mTOR-S2448-positive in both control and Rapamycin group (Figure 8).

We next examined indicators of mTORC1 signaling, including changes in the levels of phospho-S6-ribosomal protein (S6)-Ser235-236 (pS6-S235-236) and pS6-S240-244, as well as phosphorylated form of elongation repressor eukaryotic translation initiation factor 4E-binding protein 1 (4EBP1) at Threonine-70 (p-4EBP1-T70). Despite absence of frank p-mTOR-S2448 staining, all HB in controls showed staining for all three downstream effectors of mTORC1 signaling, which ranged from intensively positive subpopulation of cells within tumor nodules to a more uniform staining of the entire foci (Figure 8). Rapamycin treatment completely abolished the presence of any of these downstream markers of mTORC1 in any of the remnant foci observed after 5 weeks of treatment (Figure 8). This shows that Rapamycin successfully decreased mTORC1 activation and hence may have decreased HB burden by affecting protein synthesis and ribosomal biogenesis, thus affecting tumor cell proliferation.

## DISCUSSION

In this study, we show that our murine model of HB using SB-HTVI delivery of mutant Yap1 and β-catenin shows a strong correlation in gene expression patterns with two independent patient HB cohorts, and also shows an enrichment in genes associated with less well-differentiated, more proliferative HB. This genetic analysis matches the mixed histological features observed in control HB tumors, which range from embryonal to crowded fetal histology based on expression of specific molecular markers like Sox9 and Cyp2e1 [9, 19]. Importantly our data supports the clinical relevance of our mouse model as a tool to study HB pathogenesis, particularly among more aggressive tumors that may be more difficult to treat [7, 19, 22].

We also show that Rapamycin treatment reduced HB and overall tumor burden in the Yap1-β-catenin model by dramatically affecting their growth supporting the its relevance in the therapy for this tumor type. This study validates the activation of mTORC1 in HB as was reported recently [14]. Liu and colleagues showed that mTORC1 is activated in HB cell lines and in the HB occurring in the Yap1-β-catenin model. They demonstrated that an mTOR inhibitor MLN0128 significantly inhibited human HB cell growth *in vitro*. The *in vivo* relevance of the mTOR signaling pathway was shown by disruption of Raptor, a positive regulator and component of the mTORC1 complex, which resulted in delayed Yap1-β-catenin-induced HB development in mice. Our study used a clinically relevant therapeutic agent to inhibit mTOR signaling in the Yap1-β-catenin HB model and showed directly its efficacy to notably reduce the HB burden following 5 weeks of treatment, which was well tolerated by the mice.

The mechanism of mTORC1 activation in HB development may be multifactorial. Liu and colleagues showed increased expression of an amino acid transporter SLC38A1 in HB, and amino acid deprivation led to mTORC1 suppression in HB cell lines [14]. Silencing of Yap1 or its paralog, the transcriptional co-activator with PDZ binding motif (TAZ), decreased SLC38A1 expression as well as mTORC1 activation in HB cells [14]. In this study, in the Yap1-β-catenin model specifically, Yap1 was shown to upregulate expression of the glutamine transporter SLC38A1, contributing to mTORC1 activation by altering glutamine levels. Yap1 has also been shown to activate mTOR signaling by downregulating PTEN, a negative regulator of mTOR [24]. Our lab has also identified that β-catenin, through its regulation of GS expression, can regulate intracellular glutamine levels and in turn induce mTORC1 activation (in revision, manuscript in review). Although established tumors in the Yap1-β-catenin HB tumor model are GS-negative, earliest nodules in this model are GS-positive and hence mTORC1 activation may be contributed by the presence of GS during early stages of tumorigenesis. Nevertheless, multiple mTORC1 activation seems to be critical in HB sustenance and growth, downstream of Yap1 and β-catenin.

Multiple mTOR inhibitors are currently approved for clinical use as immunosuppressants and have proven effective for certain solid tumors. Although most solid tumors have been shown to be sensitive to mTOR inhibition *in vitro* or in preclinical models, the clinical utility of mTOR inhibition has proven limited due to the complexity of mTOR signaling and the development of resistance [25, 26]. Rapamycin and its analogs are mostly cytostatic, significantly decreasing proliferation but not promoting tumor cell death, and as such they have shown more promise in combination with cytotoxic chemotherapies [25]. We have observed similar effects in our study, as mice 10 weeks post-HTVI treated with 5 weeks of Rapamycin diet show a similar tumor burden histologically as mice 5 weeks post-HTVI, which corresponds to the time point when Rapamycin treatment was begun. Our results show that Rapamycin had a powerful effect in slowing down HB tumor growth and significantly reducing cell proliferation, which could prove useful to potentiate the effects of other chemotherapies. Notably, most of the Rapamycin-resistant HB tumors which persist in treated mice display a more well-differentiated HB-like histology. Indeed, these tumors were absent for Sox9 and positive for Cyp2e1. Two possibilities can explain the presence of remnant disease. One is that by slowing tumor proliferation by altering cell metabolism, Rapamycin promoted differentiation of embryonal or crowded fetal HB to a more differentiated HB which are relatively indolent. The second possibility is that due to some pre-existing heterogeneity in subtypes of HB in the Yap1-β-catenin model, which displays predominantly crowded and embryonal and occasional fetal HB, Rapamycin abolishes growth and development of only embryonal and crowded fetal HB over time, whereas a more fetal HB subset persists during the course of treatment. The mechanism of differential response of more undifferentiated HB to mTORC1 suppression and more resistance of differentiated HB to Rapamycin remains under investigation.

Finally, our study also underscores the utility of US imaging for studies of liver cancer especially in the relevant animal models. While standard US imaging offers an overview of tumor burden, 3D-US provides more detailed and accurate measures of tumor burden that correlate well with the standard pathology measure of liver weight to body weight ratio. Monitoring tumor growth in the same mice over time not only reduces the number of animals needed for each experiment but also provides a better understanding of how treatments can alter the kinetics of tumor growth. Indeed, endpoint LW/BW measurements would have shown only a significant decrease in tumor burden between control and Rapamycin-treated groups. However, US data along with histological analysis demonstrated that tumors are not completely absent, but in fact small foci of a different histology in fact persist following Rapamycin in the Yap1-β-catenin HB model. One caveat remains the sensitivity of detection for very small tumors, which is evident when performing US on mice at 5 weeks post-HTVI. At this time, the gross tumor burden appears to be negligible, but histopathology shows that the liver is littered with very small nodules below the threshold of US detection. Thus, US is best used in combination with histopathology and can offer an excellent view of tumor development once tumors have grown to greater than 0.5 mm in diameter, which is small enough to provide excellent detail in the mouse liver.

## MATERIALS AND METHODS

### Microarray data analysis

Mice were sacrificed at 7, 9, or 10 weeks after Yap1-β-catenin SB-HTVI for extraction of livers with significant HB tumor burden. Tumor-bearing livers (n=3) and normal livers from non-injected mice (n=3) were utilized for mRNA isolation and analyzed using Affymetrix gene array chip R430 2.0. The full data set is available at Gene Expression Omnibus (http://www.ncbi.nlm.nih.gov/geo, accession number GSE112485). The raw CEL files were imported into R (version 3.5.0) using the *affy* package [27]. Probes were mapped to genes using the custom brain array CDF [28]. The *gcrma* package was used to perform GCRMA (Guanine Cytosine Robust Multi-Array Analysis) normalization [29]. Low expressed genes were then filtered out using *genefilter* by selecting only genes with an expression value of 3 or more in at least 3 samples [30]. Gene annotation information was added using the *annotate* and *mouse4302.db* packages [31, 32]. Principal component analysis was performed in base R and the results were plotted using ggplot2 and ggfortify [33, 34]. Next, the *limma* package was used to apply an empirical Bayes statistical model to calculate a moderated t-statistic and p-value for each gene comparing its log-fold expression in HB samples relative to the WT samples [35]. Using an adjusted p-value cutoff of 0.05, 3263 differentially expressed genes were identified. The processed gene data set was then uploaded into BaseSpace Correlation Engine (which correctly identified 3106 genes for further analysis) and compared with a large database of previously published and curated gene expression data sets to identify sets with significant overlap [21]. In addition, the processed gene expression values for all genes were uploaded into Gene Set Enrichment Analysis (GSEA) software (version 3.0) for comparison with curated gene sets using 1000 gene-set permutations; for genes associated with multiple probe sets, the median of the expression values was used for analysis [20, 36].

### Animals, plasmids, and treatment

All animal experiments were approved by the Institutional Animal Use and Care Committee at the University of Pittsburgh, School of Medicine. FVB/N mice were purchased from Jackson Laboratory (Bar Harbor, ME). Hydrodynamic tail vein injections (HTVI) were performed as described before [12]. A mix of 10μg pt3-EF1α-YAPs127a, 10μg pt3-EF1α-ΔN90-β-catenin together with 4μg pcmv/SB was injected into the mice. 16 mice were randomized into two groups. The control group was kept on normal diet for 10 weeks post-HTVI (n=8), while the treated group was fed normal diet for 5 weeks post-HTVI, then treated with diet containing 19 mg/kg Rapamycin (Research Diets, Inc.) for another 5 weeks (n=8). All 16 mice were sacrificed at 10 weeks post HTVI. An additional two mice kept on normal diet were sacrificed 8 weeks post-HTVI to compare US imaging with gross pathology.

### Sonographic examination

Ultrasound (US) scans were performed on each mouse at 4, 7, 8, 9, and 10 weeks to evaluate liver volume and liver tumor. US was performed using a Visual Sonics Vivo 3100 scanner (Fujifilm, Japan) equipped with a transducer (MX250S) with 20MHz central frequency 3D capability. Mice were stabilized on a warm operating table and sedated using an inhalation anesthesia system loaded with a mixture of isoflurane and oxygen. The transducer mounting system was employed to fix the transducer in a longitudinal position perpendicular to the mouse body. First, the liver and tumors were observed carefully using the standard 2D-US imaging. Next, the US was switched to a 3D mode and scanning was begun with the transducer at the left side of gall bladder and scanned in a longitudinal direction. The following parameters were used for the 3D-scan: transducer frequency 21MHz, output power 100%, gain 23∼27dB, dynamic range 60dB, depth 18 mm, 3D range 27∼35mm, and 3D step size 0.1 mm. 3D scans were stored in the hard drive of the scanner and transferred to the software system installed in a computer for analysis later.

US scan and imaging analysis was performed by a trained radiologist with experience in diagnosis and treatment of human liver tumors (H.Y.). VisualSonics Vivo LAB 3.0.0 software (Fujifilm, Japan) was used to measure liver volume and tumor diameter. Multi-slice method was employed to measure the total liver volume; the boundary of liver on each sectional slice was delineated and the 3D liver image was reconstructed to calculate liver volume by software analysis. Liver tumors were then identified on sequential sectional images frame by frame, and the maximum tumor diameter for each lesion was measured on the largest cross-sectional slice of the targeted lesion. The volume of each tumor was then approximated as the volume of a sphere using the maximum tumor diameter for each lesion and was calculated by mathematical formula: 4/3 πr^3^ (r=tumor diameter/2). Based on the high correlation of measured total tumor volumes with liver weight to body weight ratio, this approximation was deemed sufficient for the present study.

### Immunohistochemistry (IHC)

Livers were harvested and fixed in 10% formalin for 48 hours, followed by paraffin embedding. For H&E, 4μm paraffin sections were deparaffinized and rehydrated, stained with Shandon’s Hematoxylin (Thermo-Fisher, 7211) for 45 seconds and Eosin (Thermo-Fisher, 71204) for 15 seconds, dehydrated, and mounted using Cytoseal XYL (Thermo-Fisher, 8312-4). For immunohistochemistry, deparaffinized sections were either microwaved for 12 minutes in pH6 sodium citrate buffer (Myc-tag, Cyp2E1, Cyclin D1, phospho-mTOR Serine-2448 or S2448, phospho-S6 ribosomal protein S235/236, phospho-S6 ribosomal protein S240/244, and phospho-4EBP1 Threonine 70 or T70), microwaved for 8 minutes in 1% zinc sulfate buffer (PCNA), or were pressure cooked for 20 minutes in pH 6 sodium citrate buffer (Yap1, Sox9) for antigen retrieval. Next, slides were treated with 3% hydrogen peroxide to inactivate endogenous peroxidases, and blocked with Superblock (Scytek Laboratories, AAA500). Sections were incubated overnight at 4C in the following primary antibodies: Yap1 (1:50; Cell Signaling CS14074), Sox9 (1:2000, EMD Millipore ab5535). Alternatively, sections were incubated for one hour at room temperature in the following primary antibodies: Myc-tag (1:100; Maine Medical Center Research Institute Vli01), PCNA (1:100; Santa Cruz Biotechnology sc-56), Cyclin D1 (1:200; Abcam 134175), GS (1:2000; Sigma G2781), Cyp2E1 (1:100; Sigma HPA-0009128), phospho-mTOR Ser 2448 (1:100; Cell Signaling, 2976), phospho-S6 ribosomal protein Ser 235/236 (1:50; Cell Signaling, CS4858), phospho-S6 ribosomal protein Ser 240/244 (1:50, Cell Signaling, CS5364), and phospho-4EBP1 Thr 70 (1:50; Cell Signaling CS9455). Sections were then incubated for 30 minutes at room temperature with biotin-conjugated secondary anti-mouse, -rabbit or -goat antibodies (Vector Laboratories), and developed using the VECTASTAIN ABC HRP kit (Vector Laboratories, PK-6101) and Vector DAB kits (Vector Laboratories, SK-4100). Sections were counterstained in Shandon’s Hematoxylin, dehydrated, and mounted in DPX. Images were taken on a Zeiss Axioskop 40 inverted brightfield microscope. Images for tiling were taken on a Zeiss Axio Observer. Z1 microscope and assembled utilizing ZEN Imaging software.

## SUPPLEMENTARY MATERIALS FIGURE


