# Correction: mTOR inhibition affects Yap1-β-catenin-induced hepatoblastoma growth and development

**DOI:** 10.18632/oncotarget.27218

**Published:** 2019-09-24

**Authors:** Laura Molina, Hong Yang, Adeola O. Adebayo Michael, Michael Oertel, Aaron Bell, Sucha Singh, Xin Chen, Junyan Tao, Satdarshan P. Monga

**Affiliations:** ^1^ Division of Experimental Pathology, Department of Pathology, University of Pittsburgh School of Medicine, Pittsburgh, PA, USA; ^2^ Medical Scientist Training Program, University of Pittsburgh School of Medicine, Pittsburgh, PA, USA; ^3^ Department of Medical Ultrasonics, The First Affiliated Hospital of Guangxi Medical University, Guangxi, China; ^4^ Department of Biomedical Engineering, Georgia Institute of Technology, Atlanta, GA, USA; ^5^ Pittsburgh Liver Research Center, University of Pittsburgh Medical Center and University of Pittsburgh School of Medicine, Pittsburgh PA, USA; ^6^ Department of Bioengineering and Therapeutic Sciences and Liver Center, University of California, San Francisco, CA, USA; ^7^ Division of Gastroenterology, Hepatology and Nutrition, Department of Medicine, University of Pittsburgh School of Medicine, Pittsburgh, PA, USA

**This article has been corrected:** The correct author information is given below:

**Satdarshan P. Monga**

Original article: Oncotarget. 2019; 10:1475–1490. 1475-1490. https://doi.org/10.18632/oncotarget.26668

