# Supplementary material for: mTOR inhibition affects Yap1-β-catenin-induced hepatoblastoma growth and development
Source: Oncotarget. 2019 Feb 19;10(15):1475–90. doi: 10.18632/oncotarget.26668 (PMC6407673; doi:10.18632/oncotarget.26668)
Supplement: Supplementary file 1 [file oncotarget-10-1475-s001.pdf]

mTOR inhibition affects Yap1-β-catenin-induced hepatoblastoma growth and development

SUPPLEMENTARY MATERIALS

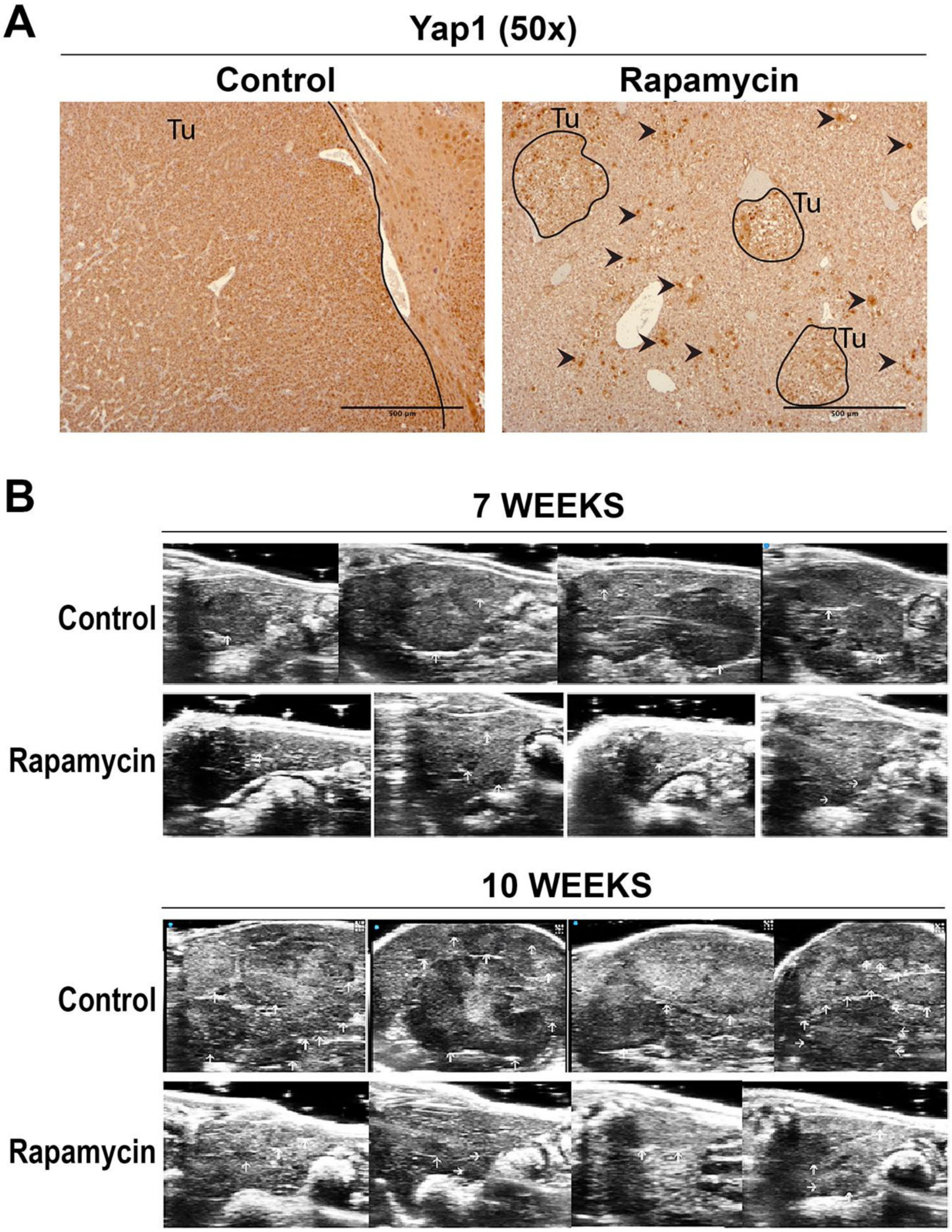

Supplementary Figure 1: (A) Representative immunohistochemistry for YAP1 showing strong nuclear staining in HB tumors in both control and Rapamycin-treated mice. **(B)** Representative ultrasound panels for control and Rapamycin-treated mice at 7 and 10 weeks. Arrows point to identified tumors in each panel.
